# Isolation and characterisation of two epithelial-like cell lines from the gills of *Chrysophrys auratus* (Australasian snapper) and *Oncorhynchus tshawytscha* (Chinook salmon) and their use in aquatic toxicology

**DOI:** 10.1007/s11626-024-00941-z

**Published:** 2024-07-10

**Authors:** Björn Böhmert, Gavril L. W. Chong, Kim Lo, Michael Algie, Damon Colbert, Melissa D. Jordan, Gabriella Stuart, Lyn M. Wise, Lucy E. J. Lee, Niels C. Bols, Georgina C. Dowd

**Affiliations:** 1 The New Zealand Institute for Plant and Food Research Limited, Nelson Research Centre, 293 Akersten Street, Nelson, 7010 New Zealand; 2 The New Zealand Institute for Plant and Food Research Limited, Mt Albert Research Centre, Auckland, 1142 New Zealand; 3 Department of Pharmacology and Toxicology, School of Biomedical Sciences, University of Otago, Dunedin, New Zealand; 4 Faculty of Science, University of the Fraser Valley, Abbotsford, BC V2S 7M8 Canada; 5 Department of Biology, University of Waterloo, Waterloo, ON N2L 3G1 Canada

**Keywords:** Australasian snapper, Chinook salmon, Epithelial, Toxicology, 3,4 DCA, EROD

## Abstract

In vitro gill models are becoming increasingly important in aquatic toxicology, yet the fish gill invitrome is underrepresented, encompassing approximately 0.1% of extant species. Here, we describe the establishment and characterisation of two gill-derived, epithelial-like cell lines isolated from fish species of significant importance to New Zealand: *Chrysophrys auratus* (Australasian snapper) and *Oncorhynchus tshawytscha* (Chinook salmon). Designated CAgill1PFR (***Chrysophrys auratus,*** **gill 1**, **P**lant & **F**ood **R**esearch) and OTgill1PFR (***Oncorhynchus tshawytscha,*** **gill 1**, **P**lant & **F**ood **R**esearch), these cell lines have each been passaged greater than each 70 times over several years and are considered spontaneously immortalised. Both cell lines required serum for growth and exhibited differential responses to basal media formulations. CAgill1PFR was sensitive to low temperatures (4 °C) but replicated at high temperatures (30 °C), whereas OTgill1PFR was sensitive to high temperatures but remained viable at low temperatures, mirroring the natural environment of their host species. Immunostaining revealed expression of epithelial cell markers cytokeratin and E-cadherin, alongside positivity for the mesenchymal cell marker, vimentin. CAgill1PFR was more sensitive to the environmental toxin 3,4 dichloroaniline than OTgill1PFR through measurements of metabolic activity, membrane integrity, and lysosomal function. Furthermore, CAgill1PFR produced less CYP1A activity, indicative of ongoing biotransformation processes, in response to beta-naphthoflavone than OTgill1PFR. These cell lines expand the toolbox of resources and emphasise the need for species-specific aquatic toxicology research.

**Supplementary Information:**

The online version contains supplementary material available at 10.1007/s11626-024-00941-z.

## Introduction

Teleostei represents the largest infraclass within the class Actinopterygii, commonly known as the ray fin fishes. Comprising approximately 96% extant fish species, teleosts inhabit diverse aquatic ecosystems, including freshwater and marine environments, and serve as a significant nutrient source for humans (Nelson 1984; Wootton 1990; FAO 2022). While many of the organs of teleosts have comparable functions to those of higher vertebrates (Roberts and Ellis 2012), the respiratory system is noticeably different, requiring an organ that allows gas exchange in aquatic environments.

Teleost respiration occurs primarily through the gills, which also fulfil crucial roles in osmoregulation (Greenwell et al. 2003; Krogh 2015), endocrine regulation (Olson 1998; McCormick 2001; Aruna et al. 2021), and pH regulation (Claiborne et al. 2002; Zimmer and Perry 2022), as well as acting as a physical barrier to the external environment (Evans et al. 2005; Chen et al. 2023). Many of these functions are localised to the gill filament – blood-perfused lamellae comprised of several specialised cell types, including pavement cells, pillar cells (modified endothelial cells), mucous/goblet cells, non-differentiated cells, neuroepithelial cells, and mitochondria-rich cells (chloride cells in seawater teleost) (Wilson and Laurent 2002; Chen et al. 2023). The gill filaments provide an interface between the environment and the circulatory system. As such, they serve as frontline defence against harmful toxins, xenobiotics or infectious agents (Wilson and Laurent 2002). These external factors are of particular concern in aquaculture, where they can lead to reduced growth rates, increased susceptibility to diseases, and mortality (Ashley 2007; Cuesta 2011; Emenike et al. 2022).

In vitro culture of gill cells has emerged as a valuable model for assessing the toxicological impact of various stressors on teleost fish (Lee et al. 2009). Effects of different toxins on cell viability, functionality, morphology, attachment, proliferation, metabolism, plasma membrane integrity, and lysosomal functionality have been described for cultured fish cells (Schirmer et al. 1997; Tanneberger et al. 2013; Yue et al. 2015; Hernández-Moreno et al. 2022a, b; Solhaug et al. 2023). Biotransformation, the process of detoxifying a compound by altering its original structure, has also been investigated using in vitro models (Stadnicka-Michalak et al. 2018; Leguen et al. 2000; Ivanova et al. 2023; Slattery et al. 2023). While traditionally associated with the liver, biotransformation has been demonstrated to occur extra-hepatically in the gills (Smolowitz et al. 1992; Matsuo et al. 2008). In vitro, biotransformation is usually demonstrated by assessing the catalytic activity of CYP1A, a key enzyme in the detoxification process, through ethoxyresorufin-*O*-deethylase (EROD) activity (Franco et al. 2018; Stadnicka-Michalak et al. 2018; Ivanova et al. 2023).

One of the most commonly utilised gill models for toxicological analysis is RTgill-W1, derived from *Oncorhynchus mykiss* (rainbow trout) (Bols et al. 1994). This cell line has been used to investigate the toxic potential of several aquatic toxins including emerging contaminants benzotriazoles (Zeng et al. 2016), wastewater/effluent (Dayeh et al. 2009; Scott et al. 2023), fragrance chemicals (Natsch et al. 2018), mycotoxins associated with fish feed (Bernal-Algaba et al. 2021), polystyrene and polycyclic aromatic hydrocarbons (PAHs) (Bussolaro et al. 2019), nanoparticles (Yue et al. 2015, 2017; Jimeno-Romero et al. 2021), and nanobiomaterials (Hernández-Moreno et al. 2022a, b). RTgill-W1 has also been used to develop a standardised method for determining acute toxicity (Schirmer 2006; Schirmer et al. 2008; Fischer et al. 2019). Biotransformation of benzo[a]pyrene (BaP) or B-naphthoflavone (BNF) through CYP1A activity could not be detected in RTgill-W1. Despite this, there was rapid loss of BaP in the culture system over time suggesting transformation was occurring via a different route, albeit slower than cells derived from the rainbow trout liver (RTL-W1) or gut (RTgutGC) (Franco et al. 2018; Stadnicka-Michalak et al. 2018; OECD 2021).

Gill cell lines from other species have also been employed in toxicological analysis. The cell line, PGGG derived from a *Epinephelus lanceolatus* × *Epinephelus fuscoguttatus* hybrid (pearl gentian grouper), has been used to investigate the effect of the heavy metal cadmium on cytotoxicity and oxidative stress (Xu et al. 2021). Gill lines LRG and CSG from *Labeo rohita* and *Channa striatus*, respectively, have been used to investigate the effect of insecticides malathion and endosulfan (Abdul Majeed et al. 2013, 2014). Together with ICG, a gill line derived from *Catla catla*, LRG has also been used to demonstrate the cytotoxicity, genotoxicity, and oxidative stress caused by silver nanoparticles (Taju et al. 2014). Atlantic salmon line ASG-10 has been employed to investigate the effect of the insecticide rotenone on reactive oxygen species production (Solhaug et al. 2023). ASG-10 also expresses CYP1A in response to benzocaine, a local anaesthetic commonly used in aquaculture (Ivanova et al. 2023). ASG-10 has also been shown to express several additional biotransformation genes including *cyp51, cyp20a1,* and *pora* (Slattery et al. 2023).

The fish gill invitrome (i.e. a grouping of cell lines around a theme or category), consists of 40 cell lines from 25 species (Bols et al. 2017, 2023; Bairoch 2018), representing 0.1% of all fish species (including cartilaginous, bony fishes, and jawless fishes). However, much like whole fish, fish cells isolated from different species respond differently to environmental stresses ( Zurita et al. 2019; Fleurbaix et al. 2022; Solhaug et al. 2023). Therefore, species-specific toxicological tools are important, particularly for aquaculture species. Two species lacking representation in the gill invitrome are *Oncorhynchus tshawytscha* (Chinook salmon) and *Chrysophrys auratus* (Australasian snapper), two important commercial fish species for New Zealand. Chinook salmon are New Zealand’s only existing aquaculture finfish species, contributing NZ$170 million to the country’s export earnings (Aquaculture New Zealand 2024). Snapper, one of New Zealand’s largest inshore fisheries, is being explored as a potential aquaculture species. A selective breeding programme to produce domesticated snapper strains with enhanced growth and survival is ongoing in New Zealand (Ashton et al. 2019; Wellenreuther et al. 2019; Moran et al. 2023). Here we report on two cell lines established from snapper and Chinook salmon gill tissues. We have designated these cell lines CAgill1PFR (***Chrysophrys auratus***, **gill** 1, **P**lant & **F**ood **R**esearch—species; tissue; institute affiliation) and OTgill1PFR (***Oncorhynchus tshawytscha***, **gill 1, P**lant & **F**ood **R**esearch). Our findings show that these cell lines are epithelial in origin and are useful models for toxicological analyses.

## Materials and methods

### Cell isolation and primary culture

A single healthy juvenile Australasian snapper (*Chrysophrys auratus,* herein called snapper*;* 2 g, 5 cm) bred at The New Zealand Institute for Plant and Food Research Limited (PFR) and a single healthy Chinook salmon *(Oncorhynchus tshawytscha*; 730 g, 32 cm) from Salmon Smolt New Zealand (Kaiapoi, New Zealand) were euthanised with an overdose of anaesthetic (60 ppm of AQUI-S®; AQUI-S Ltd, Lower Hutt, New Zealand). Each fish was surface sterilised with 70% ethanol and the gill arches removed. Following extensive washing with Hanks’ balanced salt solution (HBSS, Sigma-Aldrich, St Louis, MO) supplemented with 100 units mL^−1^ of penicillin and 100 µg mL^−1^ streptomycin (1 × P/S, Thermo Fisher, Waltham, MA), the gill lamellae were minced into small fragments using a sterile scalpel blade. Tissue fragments from snapper were immediately transferred to a 25 cm^2^ cell culture flask (Nunc™ EasYFlask™, Thermo Fisher) containing 2 mL of Leibovitz’s L-15 (L-15, Gibco, Grand Island, NY) medium supplemented with 10% foetal bovine serum (FBS, Gibco) and 1 × P/S (Thermo Fisher). Gill fragments from salmon underwent additional enzymatic dissociation with 1 mg mL^−1^ type 1a collagenase (Sigma-Aldrich) diluted in Dulbecco’s phosphate buffered saline (DPBS, Thermo Fisher) at room temperature for 45 min. The dissociated tissue fragments were centrifuged at 95 × g for 5 min (Hettich 320R), the supernatant discarded, the pellet resuspended in fresh media (L-15, 10% FBS, 1 × P/S), and divided equally between two 25 cm^2^ cell culture flasks (Falcon, Corning, NY). Regular partial or complete media changes with L-15, 10% FBS, 1 × P/S were carried out on snapper and salmon cells until the cultures reached confluency. Cells were then detached using 1 × TrypLE Express (Gibco), split between two new 25cm^2^ flasks, and continually passaged. The resulting cell lines were identified as CAgill1PFR and OTgill1PFR.

### Cell line maintenance

CAgill1PFR and OTgill1PFR were routinely cultured in 75 cm^2^, non-vented cell culture flasks (Nunc™ EasYFlask™, Thermo Fisher) at 18 °C in the dark. L-15 supplemented with 10% FBS and 1 × P/S (Sigma-Aldrich) was used as routine culture medium. Every 5–7 days, sub-confluent (90%) to fully confluent cell monolayers were detached from the culture flask using 1 × TrypLE Express (Gibco) for up to 7 min. The resulting cell suspension was centrifuged at 95 × *g* for 5 min. The spent media was discarded, and the cell pellet resuspended in fresh media and split evenly between two fresh 75 cm^2^ flasks (10 mL total media per flask). For cryopreservation, 5 × 10^6^ cells in total were pelleted (95 × *g*, 5 min) and resuspended in 0.5 ml L-15 supplemented with 20% FBS. An equal volume of freezing media (L-15 with 20% dimethyl sulfoxide (DMSO, Sigma-Aldrich)) was added, gently mixed, and instantly transferred to − 80° in a CoolCell® freezing container (Corning, NY). Frozen cells were stored at − 80 °C for at least 48 h before long-term storage in the vapour phase of liquid nitrogen. Cells were resuscitated by rapid thawing, followed by addition to 10 mL L15, 10% FBS, 1X P/S in a plugged T75 flask and incubated in the dark at 18 °C.

### Mycoplasma detection

CAgill1PFR at passage 33 and OTgill1PFR at passage 17 were cultured to confluency in the absence of antibiotics for 7 days and analysed for mycoplasma species using the LookOut® Mycoplasma PCR Detection Kit (Sigma-Aldrich) according to the manufacturer’s instructions.

### Species provenance

DNA barcoding was carried out as described by Chong et al. (2022). Briefly, conserved sequences of cytochrome oxidase subunit I (*COI)* and 16S rRNA were amplified from CAgill1PFR and OTgill1PFR and the sequences compared with sequenced amplicons of the same genes from gDNA extracted from the fin of snapper and Chinook salmon, respectively.

### Effects of FBS, basal media, and temperature on growth of gill cell lines

MTT ((3-(4,5-Dimethylthiazol-2-yl)-2,5-Diphenyltetrazolium Bromide) metabolism by cells is routinely used as a proxy for cell proliferation. Preliminary experiments confirmed the correlation between cell number (by trypsinisation of cells and counting by haemocytometer) and MTT metabolism (data not shown). As a result, MTT assays were used to determine the impact of different culture parameters on cell growth.

OTgill1PFR (6 × 10^4^ cells well^−1^) and CAgill1PFR (4 × 10^4^ cells well^−1^) were seeded in 24-well plates (Nunclon™ Delta treated, Thermo Fisher) containing 1 mL of culture medium (L-15, 10% FBS 1 × P/S). Plates were incubated at 18° C to allow cells to adhere to the well surface (Day 0). Twenty-four hours later, cells were challenged with varying culture conditions including different concentrations of foetal bovine serum (0%, 5%, 10%, 15%, or 20%), different temperatures (4 °C, 12 °C, 20 °C, or 30 °C), or different basal media formulations (L-15, CO_2_ Independent Medium (CIM, Gibco), Medium 199-HBSS (M199-HBSS, Gibco), MEM-Hanks’ Balanced Salts (MEM-HBSS, Gibco)). 1 × P/S was added to every culture condition and 10% FBS was used for each temperature and basal media condition. Test media was replaced seven days post seeding.

Cells were monitored microscopically (Olympus CKX53) and MTT assays carried out on days 1, 4, 7, and 10 as described by Chong et al. (2022). Briefly, cells were washed with DPBS twice and incubated in 0.4 mL of serum- and antibiotic-free L-15 with 0.1 mL MTT (5 mg mL^−1^) (no FBS/P/S) in the dark at 18 °C. Seventy minutes later, 0.5 mL of DMSO was added to each well and the plate placed on a rocking shaker for 10 min, followed by gentle trituration to ensure complete solubilisation of the purple formazan crystals. Subsequently, 0.9 mL from each well was transferred to a cuvette and its absorbance measured at 600 nm (Spectrometer ThermoScientific Evolution220).

Three biological replicates, each comprising three technical replicates were carried out for each test condition.

### Immunocytochemistry

Cells were seeded onto acid-washed coverslips (Pure Science, Wellington, New Zealand) in a 24-well plate and allowed to adhere overnight (CAgill1PFR 2.5 × 10^5^ cells well^−1^ and OTgill1PFR 3 × 10^5^ cells well^−1^). Cells were washed briefly with L-15/Ex (protein free solution containing salts, galactose, and pyruvate (Schirmer et al. 1997)) then fixed in well with 4% formaldehyde in PBS for pan cytokeratin, fibronectin and vimentin labelling. For e-cadherin detection, cells were fixed in an ice-cold 1:1 mix of acetone:methanol for 15 min at room temperature to preserve junctional complexes as described by Buckley et al. (2018). Formaldehyde-fixed cells were permeabilised in 0.1% Triton X-100 (Sigma-Aldrich) in PBS for 15 min at room temperature.

Cells were rinsed once in wash buffer (0.1% bovine serum albumin (BSA, Sigma-Aldrich) in PBS), followed by blocking of non-specific antibody binding with 1% BSA (Sigma-Aldrich) in PBS (blocking buffer). Primary antibodies targeting cytokeratin (mouse anti-pancytokeratin, PCK-C2562 monoclonal Sigma-Aldrich), E-cadherin (rabbit anti-Cdh1 polyclonal, GTX125890 GeneTex), fibronectin (mouse anti-fibronectin IST-3 monoclonal, F0791, Sigma-Aldrich), and vimentin (rabbit anti-Vim polyclonal, GTX133061, GeneTex) were diluted 1:100 in blocking buffer and incubated with the cells for 2 h at room temperature after which the cells were washed three times in wash buffer. Fluorescently labelled secondary antibodies (donkey anti-mouse IgG (H + L) Alexa Fluor™ 488, A21202, Thermo Fisher or donkey anti-rabbit IgG (H + L) Alexa Fluor™ 488, A21206, Thermo Fisher) were diluted 1:1000 in blocking buffer and incubated with the cells for 1 h at room temperature in the dark. Controls consisted of cells exposed to only the secondary antibodies. Nuclei were counterstained with NucBlue™ Fixed Cell ReadyProbes™ Reagent (Thermo Fisher) for 10 min at room temperature. Coverslips were mounted onto slides using Prolong™ Gold Antifade Mountant (Thermo Fisher).

Images were taken on an Eclipse Ni-E (Nikon, Tokyo, Japan) compound microscope using the GFP filter wheel; Ex 484–504 nm, emission 516–556 nm and UV filter cube: excitation 330–380 nm, emission 410 + nm.

Methods employed for immunohistochemistry of gill tissue are described in Supplementary Material.

### Response of gill cells to toxicants

The response of CAgill1PFR and OTgill1PFR to the model environmental toxin, 3,4-dichloroaniline (3,4 DCA) (Sigma-Aldrich) was carried out as described by Tanneberger et al. (2013). Briefly, 3.5 × 10^5^ cells were seeded in each well of a 24-well plate with 1 mL L-15 (10% FBS, 1 × P/S). Twenty-four hours later, the confluent monolayers were exposed to increasing concentrations of 3,4 DCA (6.25 mg mL^−1^, 12.5 mg mL^−1^, 25 mg mL^−1^, 50 mg mL^−1^, and 100 mg mL^−1^) in L-15/ex. 0.5% DMSO acted as a control. Plates were incubated in the dark for 24 h at 18 °C after which three metrics of cell health were recorded – the effect on cell metabolism was measured by AlamarBlue™ (AB, Thermo Fisher), the effect on cell membrane integrity was determined by 5-carboxyfluorescein diacetate acetoxy-methyl ester (CFDA-AM, Thermo Fisher) and the effect on lysosomal membrane integrity was measured by Neutral Red (NR, Sigma-Aldrich). Cells that had been exposed to 3,4 DCA were washed with L-15/ex and subsequently incubated with an AB/CFDA-AM mix for 30 min at 18 °C (5% AlamarBlue™, 0.1% 4 µM CFDA-AM in L-15/ex). The resulting fluorescence was then measured (excitation/emission—530/595 nm AB and 493/541 nm CFDA-AM; CLARIOstar, BMG Labtech). The AB/CFDA-AM solution was replaced with 0.4 mL NR working solution (1.5% (v/v) in L-15/ex) and the plates incubated in the dark for 60 min at 18 °C. The NR working solution was discarded, and 0.4 mL fixative (5 g L^−1^ CaCl_2_ (Sigma-Aldrich), 0.25% formaldehyde (37% (w/v), Sigma-Aldrich) in water) added to each well. The fixative was replaced with 0.4 mL extraction solution (1% acetic acid (Sigma-Aldrich) and 50% ethanol (Sigma-Aldrich) in water), and the plates incubated for 10 min on a gently shaking plate shaker at room temperature. Thereafter, the fluorescence of NR (ex/em 540/645 nm) was measured. Three biological replicates, each comprising three technical replicates were carried out for each test condition. Dose–response curves were created using the Origin Lab nonlinear curve fitting module.

### EROD assay

CYP1A activity was detected following the method described by Ivanova et al. (2023). Briefly, cells were seeded at a density of 4.5 × 10^4^ cells per well in a 96-well plate in 0.1 mL L15, 10%FBS,1 × P/S. Cells were incubated at room temperature in the dark for 24 h, after which cells were exposed to 1, 10, or 100 nM beta-naphthoflavone. Cells treated with 0.2% DMSO acted as a control. Twenty-four hours later, 0.2 mL EROD assay medium (phenol red-free, FBS-free L-15 with 8 µM 7-ethoxyresourufin) was added to the cells. After 30 min, 0.15 mL of the supernatant was added to a black 96-well microplate and the fluorescence measured (ex/em 544/595 nm).

### Animal handling

All research carried out in this study was reviewed and approved by the animal ethics committee of Nelson Marlborough Institute of Technology in New Zealand (Application number AEC2019-PFR-01).

## Results

### CAgill1PFR and OTgill1PFR are continuous cell lines from gill tissue of *C. auratus* and *O. tshawytscha*, respectively

**Figure 1:**
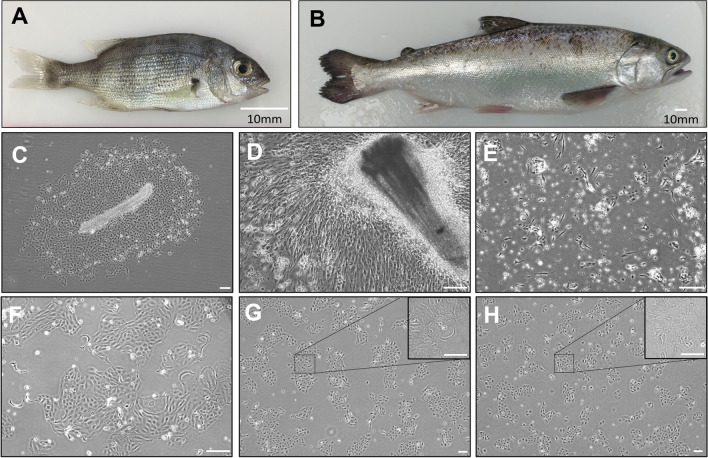
Gill cell lines CAgill1PFR and OTgill1PFR originated from (*A*) *Chrysophrys auratus* and (*B*) *Oncorhynchus tshawytscha*, respectively. Mechanical disruption of snapper gill tissue resulted in attachment of partial lamellae, with (*C*) epithelial-like cells or (*D*) fibroblast-like cells emerging. Enzymatic digestion of salmon gill tissue resulted initially in (*E*) attachment of single cells with various morphologies, which became primarily epithelial-like in early primary culture (*F*). Subconfluent (*G*) CAgill1PFR and (*H)* OTgill1PFR under routine culture conditions. Magnified culture in insert. *Scale bars* in C-H represent 100 µm.

Mechanical mincing of gills from a juvenile snapper (Fig. 1*A*) resulted in multiple tissue fragments, the majority of which appeared to be secondary lamella, attaching to the tissue culture surface. These fragments gave rise to colonies of cells with various morphologies including epithelial-like (Fig. 1*C*) and fibroblastic-like (Fig. 1*D*) cells. Salmon gills from a larger fish (Fig. 1*B*) underwent an additional enzymatic digestion and resulted in single cells that attached to the culture vessel individually, or in pairs, and grew in small clusters of cells with various morphologies (Fig. 1*E*). As the primary culture expanded, cells with a more epithelial morphology dominated the culture (Fig. 1*F*).

Through continuous passage, a single cell type from both snapper and salmon was selected for and resulted in continuous cultures, which have been passaged 143 and 68 times, respectively. Both cell lines exhibited epithelial-like morphology under phase microscopy and grew in colonies/clusters when sub-confluent. Both lines were assumed to be continuous through spontaneous immortalisation and were designated CAgill1PFR (Fig. 1*G*) and OTgill1PFR (Fig. 1*H*).

Both cell lines were cryopreserved and successfully resuscitated (~ 92–95% cell viability) in L-15 supplemented with 10% FBS. No morphological changes were observed following resuscitation. The cell lines were confirmed as being free of *Mycoplasma* species contamination (Fig. S1). Species provenance of CAgill1PFR and OTgill1PFR were verified as *Chrysophrys auratus* and *Oncorhynchus tshawytscha* by 100% homology between 16S rRNA and COI gene sequences from cell line genomic DNA and gDNA extracted from fin clips of each fish (gel images and *O. tshawytscha* sequences in Fig. S2). Sequence data for snapper can be accessed via a data repository hosted by the national infrastructure platform Genomics Aotearoa in New Zealand (https://www.genomics-aotearoa.org.nz/data). As a taonga (treasured) species to the indigenous population of New Zealand (Māori), sharing of genomic data generated from snapper must follow the principles of Māori Data Sovereignty. This enables iwi, hapū and whānau (tribes, kinship groups and families) to effectively exercise their responsibilities as guardians over biological entities that are taonga (Te Aika et al 2023).

### Culture conditions affected the replication of OTgill1PFR and CAgill1PFR

**Figure 2:**
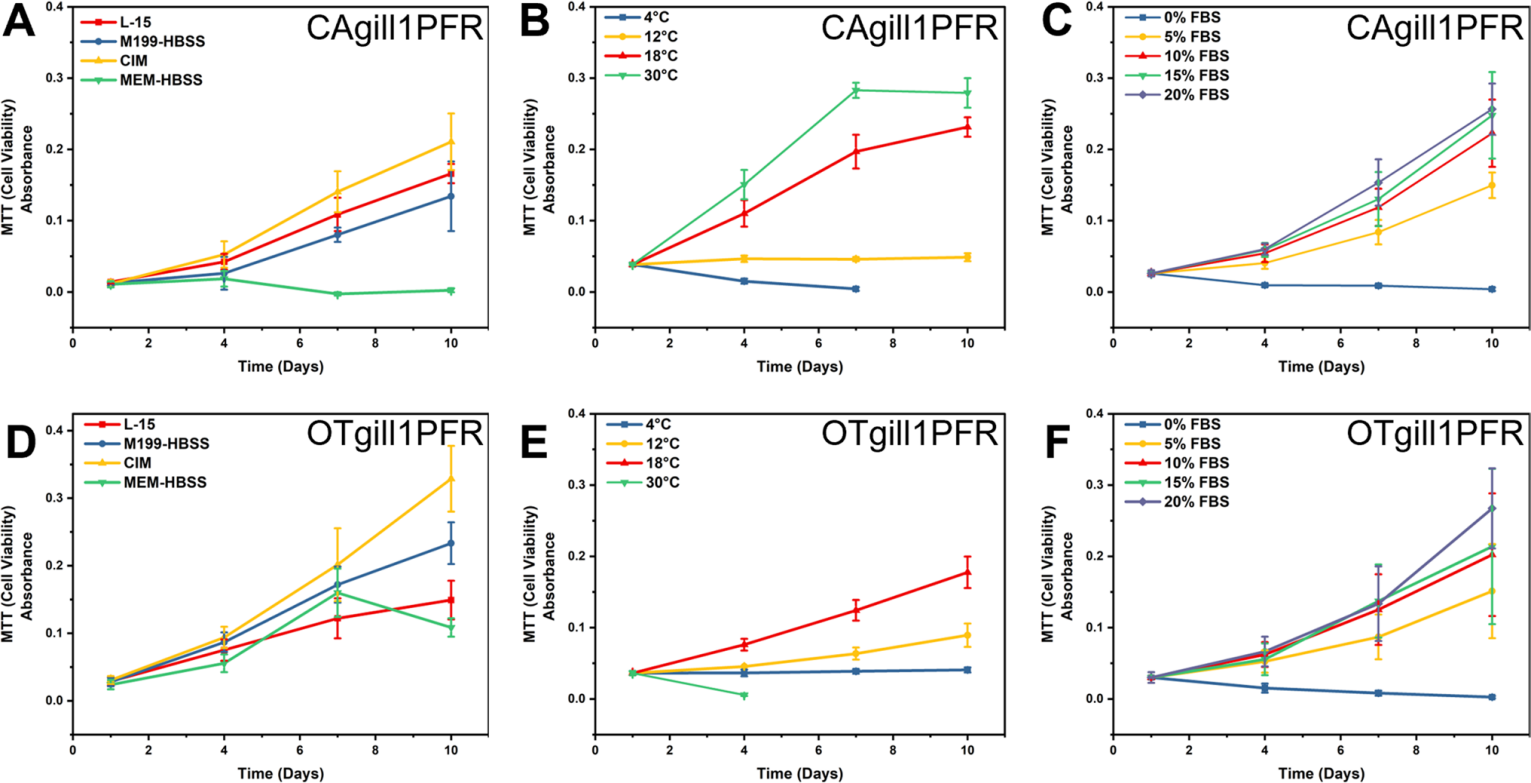
The impact of (*A*, *D*) basal media formulation, (*B*, *E*) temperature, and (*C*, *F*) FBS concentration on the metabolic activity of CAgill1PFR (*A*–*C*) and OTgill1PFR (*D*–*F*) was monitored via MTT (3-(4,5-dimethylthiazol-2-yl)-2,5-diphenyltetrazolium bromide), as a metric of cell proliferation, assay over a 10-day period. The data represent the means ± SEM of three independent experiments, each with three technical replicates.

Using MTT metabolism as a metric of cell proliferation, OTgill1PFR replication was better in CIM and M199-HBSS than in standard culture media L-15 (Fig. 2*D*); however, culture in CIM caused a change in the morphology of the cells. Cells appeared to lose the cobble-stone appearance with defined cell–cell junctions and became more loosely associated. A morphological change was also observed in CAgill1PFR where cells no longer grew in clusters/colonies, but grew individually and more dispersed throughout the culture vessel (Fig. S3*A*). MEM-HBSS was unable to support proliferation of CAgill1PFR, whereas OTgill1PFR growth was similar to that seen in the control condition (Fig. 2*A*, *D*). Culture of CAgill1PFR in M199-HBSS and CIM was comparable to the control condition (Fig. 2*A*).

CAgill1PFR replicated rapidly at 30 °C (Fig. 2*B*), whereas OTgill1PFR perished at this temperature (Fig. 2*E*). At 4 °C, OTgill1PFR remained attached to the culture vessel for the duration of the experiment (10 d) but had minimal MTT metabolism and did not appear to replicate (Fig. 2*B*). Under the same conditions CAgill1PFR detached and died off over a 7-day period (Fig. 2*E*). The effects of high (30 °C) and low temperatures (4 °C) are visible after 4 d in culture (Fig S3*B*). Both cell lines replicated at 18 °C, with a slower rate of growth at 12 °C (Fig. 2*B*, E).

Both CAgill1PFR and OTgill1PFR increased MTT metabolism/cell proliferation in response to increasing concentrations of FBS from 5 to 10%, with no further increase evident at 15–20% (Fig. 2*C*, *F*). Neither cell line was capable of growth in the absence of serum but remained attached to the culture vessel.

### OTgill1PFR and CAgill1PFR expressed epithelial cell markers

Commercial antibodies against epithelial and fibroblast cell markers were validated in excised snapper and Chinook salmon whole gill tissue (Fig. S4 and S5*A*). Pan-cytokeratin was observed around the secondary lamella of gill tissue where epithelial cells reside in both species. Pan-cytokeratin was also detected in chondrocytes located in the filament cartilage. Immunohistological staining of snapper gill tissue identified cells expressing high levels of vimentin in the secondary lamella dispersed among weaker-expressing cells. High-expression cells could also be detected at the base of the secondary lamella in some cases. In Chinook salmon gill tissue, vimentin–expressing cells were detected at the base of the secondary lamella, with weaker expression also detected throughout the lamella themselves. Isolated fibronectin positive cells were dispersed throughout the lamella of both species with significant staining in the extracellular cartilaginous matrix.

**Figure 3:**
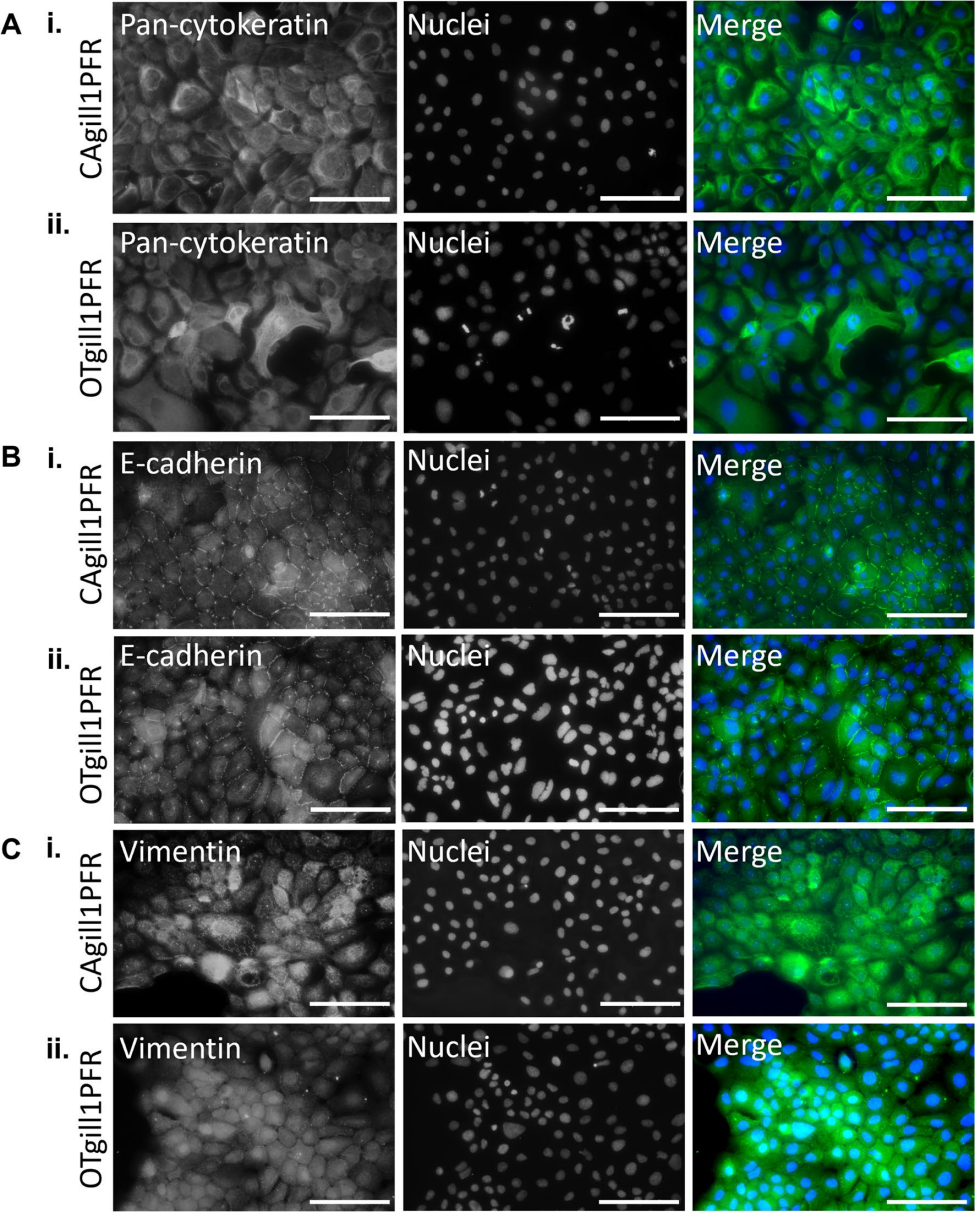
Immunocytochemistry targeting (*A*) pan cytokeratin showed fibrous cytoplasmic expression in both (*i*) CAgill1PFR and (*ii*) OTgill1PFR. (*B*) E-cadherin was detected at cell–cell junctions in (*i*) CAgill1PFR and (*ii*) OTgill1PFR. (*C*) Vimentin expression in (*i*) CAgill1PFR was visible as fibrous networks whereas expression in (*ii*) OTgill1PFR was more diffuse through the cytoplasm. *Scale bars* represent 100 µm.

OTgill1PFR and CAgill1PFR both stained positive for epithelial cell markers E-cadherin and cytokeratin (Fig. 3*A*i, ii and *B*i, ii). E-cadherin localised to the lateral junctions between cells (Fig. 3*B*). Localisation was more punctate in OTgill1PFR. Both cell lines were negative for the mesenchymal cell marker fibronectin; however, were positive for vimentin (Fig. 3*C*i, ii and Fig. S5*B*). CAgill1PFR had the expected filamentous-like structure, while OTgill1PFR vimentin labelling was more diffuse throughout the cytoplasm.

### CAgill1PFR was more sensitive to the environmental toxin 3,4 DCA than OTgill1PFR

**Figure 4:**
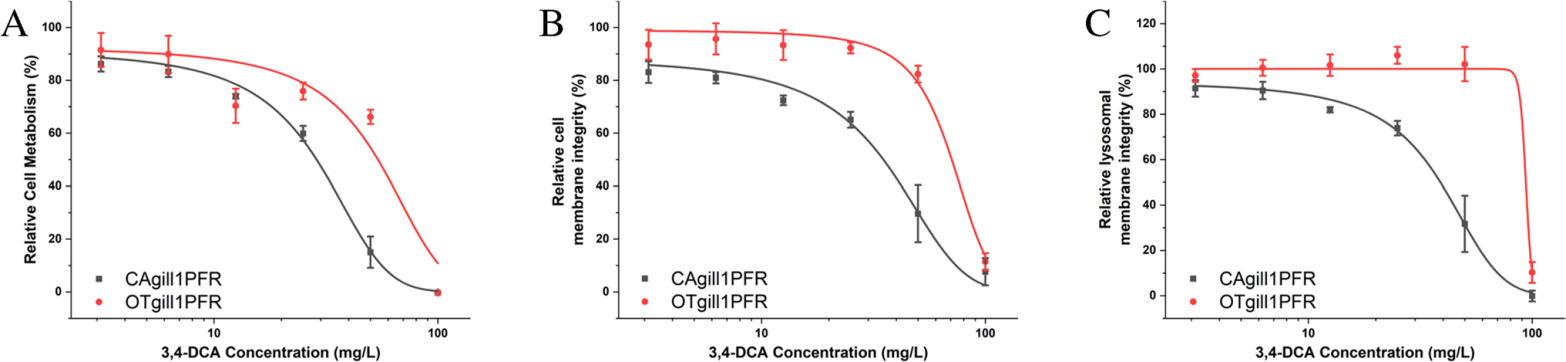
OTgill1PFR and CAgill1PFR were treated with increasing concentrations (0–100 mg/ L) of 3,4-dichloroaniline (3,4 DCA) for 24 h after which (*A*) cell metabolism was measured via Alamar blue, (*B*) membrane integrity was measured via CFDA-AM, and (*C*) lysosomal membrane integrity was measured by neutral red. The data represent the means ± SEM of three independent experiments, each with three technical replicates.

Both snapper and salmon gill cell lines exposed to increasing concentrations of the model environmental toxicant 3,4 DCA showed decreasing metabolic activity and membrane integrity, though CAgill1PFR was more sensitive to the toxin than OTgill1PFR (Fig. 4*A*, *B*). Measurements of lysosomal membrane integrity showed that salmon cells maintained the integrity consistently until a complete drop off at the highest concentration of the toxin, whereas snapper cells showed a dose-dependent response (Fig. 4*C*).

### OTgill1PFR showed greater CYP1A activity in response to *beta*-naphthoflavone than CAgill1PFR

**Figure 5:**
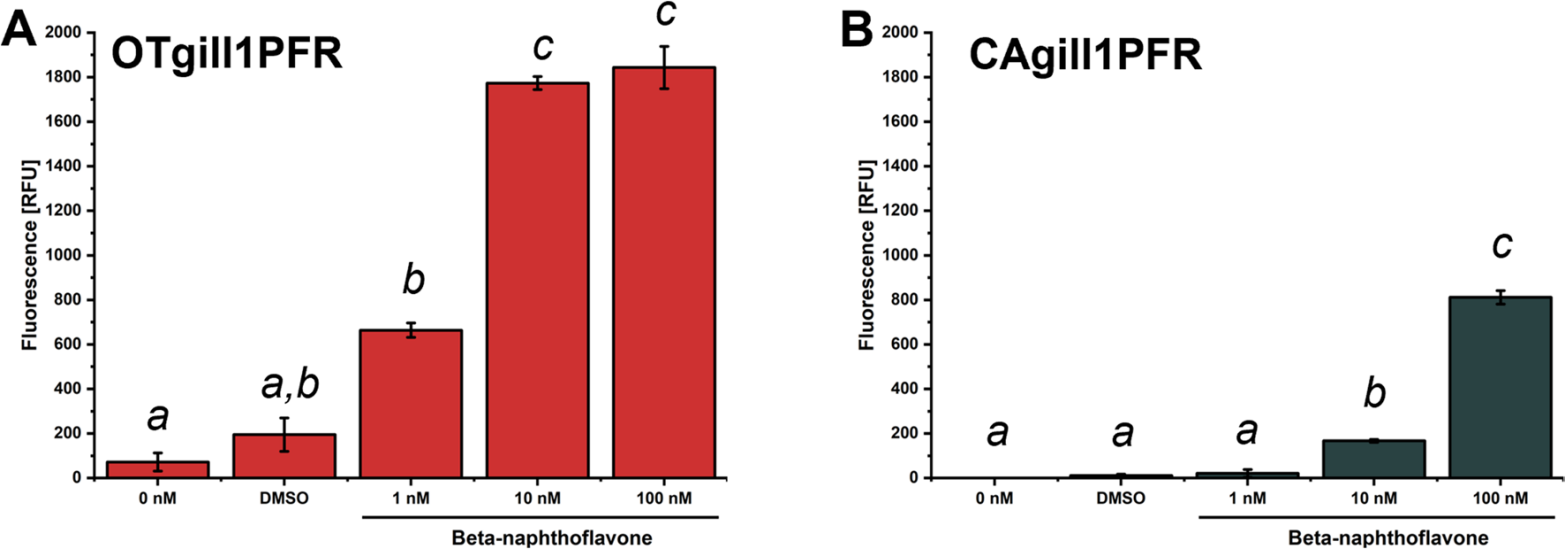
Induction of CYP1A by beta-naphthoflavone in cultures of (*A*) CAgill1PFR and (*B*) OTgill1PFR. Cultures were exposed for 24 h to increasing beta-naphthoflavone concentrations (1 to 100 nM) and CYP1A levels measured as EROD activity. The data represent the means ± SD of three independent experiments, each with three technical replicates. DMSO, the carrier for beta-naphthoflavone, acts as a control. *P* < 0.05 by one-way ANOVA followed by Tukey’s least Significant Difference test. Means that do not share a *letter* are significantly different.

When exposed to increasing concentrations of the environmental xenobiotic beta-naphthoflavone*,* activity of CYP1A, a key enzyme in biotransformation increased in both OTgill1PFR and CAgill1PFR (Fig. 5*A*, B). CYP1A activity was greater in OTgill1PFR than CAgill1PFR following beta-naphthoflavone treatment, with a significant response at the lowest concentration tested (1 nM).

## Discussion

In this study, we present the development and characterisation of two epithelial-like cell lines, CAgill1PFR and OTgill1PFR, isolated from Australasian snapper and Chinook salmon, respectively. The conditions under which OTgill1PFR and CAgill1PFR were cultured significantly affected their replication/metabolic activity, though not always in the same way. Adjusting the temperature resulted in a different response from each cell line. Notably, CAgill1PFR exhibited robust replication at 30 °C but perished at 4 °C. This temperature profile aligns closely to that of our previously reported snapper muscle-derived cell line CAtmus1PFR, indicating consistent thermal preferences across different tissue types (Chong et al. 2022). In contrast to CAgill1PFR, OTgill1PFR perished at elevated temperatures (30 °C) but survived the lower temperature (4 °C). These differential responses reflect the thermal preferences of snapper and Chinook salmon in their natural habitats (Araújo et al. 2023; Bowering et al. 2023) and underscore the utility of species-specific gill-derived cell lines as models for investigating the interplay between the extreme/abnormal temperature fluctuations induced by climate change (Pumputis et al. 2022) and other environmental stressors, like disease-causing agents and aquatic toxins.

Changing the basal media formulations caused morphological and growth pattern changes in both cell lines, highlighting the sensitivity of these cells to nutrient composition, and the importance of optimising culture conditions. An interesting finding of this study was that MEM-HBSS was found to be unsupportive of CAgill1PFR metabolism, yet this medium could support metabolism of a previously described muscle-derived cell line from the same species, CAtmus1PFR (Chong et al. 2022), and OTgill1PFR from this study. Comparing media formulations of L-15, M199-HBSS and MEM-HBSS (CIM formulation is proprietary, and is therefore omitted from this comparison), only three components present in L-15 and M199-HBSS are absent in MEM-HBSS: the non-essential amino acids serine, glycine, and alanine. It would be interesting to determine if addition of one or all three of these amino acids to MEM-HBSS restored growth of CAgill1PFR, or if additional factors are required.

The expression of epithelial cell markers E-cadherin and cytokeratin in both OTgill1PFR and CAgill1PFR is consistent with their epithelial origin, and is in agreement with the previously described Atlantic salmon gill line, ASG-10 (Gjessing et al. 2018). Vimentin, typically associated with mesenchymal-derived cells, has previously been reported to co-express with markers of other cell types such as cytokeratins (epithelial cell marker) and collagen (stromal cell marker) suggesting that vimentin expression does not necessarily restrict fish cells to a particular lineage (Herrmann et al. 1996; Markl and Franke 1988; Vo et al. 2015). Considering the complexity of vimentin expression in teleost, it is plausible that OTgill1PFR and CAgill1PFR are epithelial cells that express vimentin during usual growth. A second possibility is that OTgill1PFR and CAgill1PFR are in a culture-induced state of partial differentiation during an epithelial to mesenchymal transition (Herrmann et al. 1996; Mendez et al. 2010; Dongre and Weinberg 2019).

In vitro culture of gill cell lines is gaining momentum as a useful model for in vivo gill tissue, particularly for species in aquaculture. It offers several benefits including minimising live animal testing, cost effectiveness, controlled and high-throughput experimentation with reduced variability, as well as options for cell and species specificity. Additionally, it facilitates studies on gill development, differentiation processes, and pathophysiological responses related to disease and stress (Lee et al. 2009).

One of the most utilised applications for gill cell lines has been aquatic toxicology (reviewed in Segner 1998; Bols et al. 2005). Despite the perceived lower sensitivity of gill epithelial cells compared with live fish, there is a high correlation between the two methods supporting the validity of using cell lines in toxicological analysis (Lilius et al. 1995; Sandbacka et al. 2000; Gülden and Seibert 2005, 2007; Tanneberger et al. 2013). The robustness of gill cell models has also been demonstrated through significant inter- and intra-laboratory analyses carried out using RTgill-W1 (Fischer et al. 2019). This ultimately led to the validation of OECD Test No. 249, which is considered to be more precise and effective than whole animal trials (Burden et al. 2020; OECD 2021; Paparella et al. 2021).

CAgill1PFR and OTgill1PFR had differential responses in toxicological analysis. CAgill1PFR was more sensitive to the environmental toxin 3,4 DCA and had lower expression of the biotransformation enzyme CYP1A in response to beta naphthoflavone than OTgill1PFR. Differences between gill cell line responses to toxins have also been noted by Solhaug et al. (2023) who demonstrated that RTgill-W1 was more sensitive to the piscicide rotenone than ASG-10. In vivo studies comparing effects of aquatic toxins on *Chrysophrys auratus* and *Oncorhynchus tshawytscha* are minimal. However, one study investigating the effects of toxic dinoflagellates, determined that juvenile salmon were more sensitive than juvenile snapper (Shi et al. 2012). This is in contrast to our study, which raises the question as to why one cell line is more sensitive than another. The impact of culture conditions (environmental factors) on the susceptibility of cells to external stresses has been demonstrated in mammalian-derived epithelial monolayers (Cheek et al. 1988). Considering the effect adjusting culture parameters had on metabolism of OTgill1PFR and CAgill1PFR, it is plausible that culture conditions (e.g. increasing the temperature or using a different media) could cause a change in susceptibility to aquatic toxins. If cells were polarised to mimic in vivo conditions more closely (Mandal et al. 2020) would the susceptibility to toxins decrease as has been reported in human embryonic cultures (Hsiung et al. 2015)? Are specific molecular patterns at play? The presence of CYP1A orthologs involved in biotransformation in fish varies significantly. Some species, like *Cyrpinus carpi* (common carp), are predicted to express just two orthologs 1A2, and 1B1, whereas *Danio rerio* (zebrafish) express five (1A1, 1A2, 1B1, 2J2, and 3A4/50) (Matthee et al. 2023). Indeed, the historical life cycle of the host fish could have resulted in more resilient cells in culture. For example the Atlantic killifish (*Fundulus heteroclitus*) from heavily contaminated urban estuaries, has been shown to be ~ 8,000 times more resistant to normally lethal levels of pollution due to its high genetic diversity driving rapid adaption (Reid et al. 2016).

Despite these questions, our study contributes to the growing body of knowledge on fish cell culture and its applications in toxicological research. The establishment and characterisation of CAgill1PFR and OTgill1PFR cell lines provides valuable tools for studying species-specific responses to environmental stressors, creating an understanding of biotransformation pathways and detoxification mechanisms in teleost fish, and advancing our understanding of fish gill physiology and toxicology.

## Supplementary Information

Below is the link to the electronic supplementary material.

Supplementary file1 (DOCX 6.40 MB)

## Data Availability

The authors declare that the data supporting the findings of this study are available within the paper and its Supplementary Information files. Should any raw data files be needed in another format they are available from the corresponding author upon reasonable request.

## References

[CR2] Abdul Majeed S, Nambi KSN, Taju G, Sarath Babu V, Farook MA, Sahul Hameed AS (2014) Development and characterization of a new gill cell line from air breathing fish *Channa striatus* (Bloch 1793) and its application in toxicology: Direct comparison to the acute fish toxicity. Chemosphere 96:89–98. 10.1016/j.chemosphere.2013.07.045 23972731 10.1016/j.chemosphere.2013.07.045

[CR1] Abdul Majeed S, Nambi KSN, Taju G, Sundar Raj N, Madan N, Sahul Hameed AS (2013) Establishment and characterization of permanent cell line from gill tissue of *Labeo rohita* (Hamilton) and its application in gene expression and toxicology. Cell Biol Toxicol 29:59–73. 10.1007/s10565-012-9237-7 23224722 10.1007/s10565-012-9237-7

[CR3] Aquaculture New Zealand (2024) Mussels & salmon set new export records in 2023. In: Neas M (ed) Aquaculture New Zealand Magazine. Available via https://www.aquaculture.org.nz/resources/magazine

[CR4] Araújo BC, Miller MR, Walker SP, Symonds JE (2023) The influence of temperature on performance, biological indices, composition, and nutrient retention of juvenile Chinook salmon (*Oncorhynchus tshawytscha*) reared in freshwater. Comp Biochem Physiol Part A Mol Integr Physiol 280:111412. 10.1016/j.cbpa.2023.111412 10.1016/j.cbpa.2023.111412 36878388

[CR5] Aruna A, Wang TP, Cao JC, Lan DS, Nagarajan G, Chang CF (2021) Differential expression of hypothalamic and gill-crh system with osmotic stress in the euryhaline black porgy. Acanthopagrus Schlegelii Front Physiol 12:768122. 10.3389/fphys.2021.768122 34858213 10.3389/fphys.2021.768122 PMC8632050

[CR6] Ashley PJ (2007) Fish welfare: Current issues in aquaculture. Appl Anim Behav Sci 104:199–235. 10.1016/j.applanim.2006.09.001

[CR7] Ashton DT, Hilario E, Jaksons P, Ritchie PA, Wellenreuther M (2019) Genetic diversity and heritability of economically important traits in captive Australasian snapper (*Chrysophrys auratus*). Aquaculture 505:190–198. 10.1016/j.aquaculture.2019.02.034

[CR8] Bairoch A (2018) The Cellosaurus, a Cell-Line Knowledge Resource. J Biomol Tech 29:25–38. 10.7171/jbt.18-2902-002 29805321 10.7171/jbt.18-2902-002 PMC5945021

[CR9] Bernal-Algaba E, Pulgarín-Alfaro M, Fernández-Cruz ML (2021) Cytotoxicity of mycotoxins frequently present in aquafeeds to the fish cell line RTGill-W1. Toxins 13:581. 10.3390/toxins13080581 34437452 10.3390/toxins13080581 PMC8402477

[CR10] Bols NC, Barlian A, Chirino-trejo M, Caldwell SJ, Goegan P, Lee LEJ (1994) Development of a cell line from primary cultures of rainbow trout, *Oncorhynchus mykiss* (Walbaum), gills. J Fish Dis 17:601–611. 10.1111/j.1365-2761.1994.tb00258.x

[CR13] Bols NC, Dayeh VR, Lee LEJ, Schirmer K (2005) Chapter 2 Use of fish cell lines in the toxicology and ecotoxicology of fish. Piscine cell lines in environmental toxicology. In: Mommsen TP and Moon TW (eds) Biochem Mol Biol Fishes, volume 6, Environmental Toxicology, Elsevier, Amsterdam, pp 43–84. 10.1016/S1873-0140(05)80005-0

[CR12] Bols NC, Lee LEJ, Dowd GC (2023) Distinguishing between and properties of animal cell lines and demonstrating their use in grouping ray-finned fish cell lines into invitromes. In Vitro Cell Dev Bio Anim 59:41–62. 10.1007/s11626-022-00744-0 36719554 10.1007/s11626-022-00744-0

[CR11] Bols NC, Pham PH, Dayeh VR, Lee LEJ (2017) Invitromatics, invitrome, and invitroomics: introduction of three new terms for in vitro biology and illustration of their use with the cell lines from rainbow trout. In Vitro Cell Dev Biol Anim 53:383–405. 10.1007/s11626-017-0142-5 28374170 10.1007/s11626-017-0142-5

[CR14] Bowering LR, McArley TJ, Devaux JBL, Hickey AJR, Herbert NA (2023) Metabolic resilience of the Australasian snapper (*Chrysophrys auratus)* to marine heatwaves and hypoxia. Front Physiol 14:1215442. 10.3389/fphys.2023.1215442 37528894 10.3389/fphys.2023.1215442 PMC10387550

[CR15] Buckley AG, Looi K, Iosifidis T, Ling KM, Sutanto EN, Martinovich KM, Kicic-Starcevich E, Garratt LW, Shaw NC, Lannigan FJ, Larcombe AN, Zosky G, Knight DA, Rigby PJ, Kicic A, Stick SM (2018) Visualisation of Multiple Tight Junctional Complexes in Human Airway Epithelial Cells. Biol Proced Online 20:3. 10.1186/s12575-018-0070-0 29434527 10.1186/s12575-018-0070-0 PMC5793437

[CR16] Burden N, Benstead R, Benyon K, Clook M, Green C, Handley J, Harper N, Maynard SK, Mead C, Pearson A, Ryder K, Sheahan D, van Egmond R, Wheeler JR, Hutchinson TH (2020) Key Opportunities to Replace, Reduce, and Refine Regulatory Fish Acute Toxicity Tests. Environ Toxicol Chem 39:2076–2089. 10.1002/etc.4824 32681761 10.1002/etc.4824 PMC7754335

[CR17] Bussolaro D, Wright SL, Schnell S, Schirmer K, Bury NR, Arlt VM (2019) Co-exposure to polystyrene plastic beads and polycyclic aromatic hydrocarbon contaminants in fish gill (RTgill-W1) and intestinal (RTgutGC) epithelial cells derived from rainbow trout (*Oncorhynchus mykiss*). Environ Pollut 248:706–714. 10.1016/j.envpol.2019.02.066 30849588 10.1016/j.envpol.2019.02.066 PMC6794159

[CR18] Cheek JM, Postlethwait EM, Crandall ED (1988) Effects of Culture Conditions on Susceptibility of Alveolar Epithelial-Cell Monolayers to No2. Toxicol Lett 40:247–255. 10.1016/0378-4274(88)90048-3 3354009 10.1016/0378-4274(88)90048-3

[CR19] Chen XY, Liu SB, Ding QW, Teame T, Yang YL, Ran C, Zhang Z, Zhou ZG (2023) Research advances in the structure, function, and regulation of the gill barrier in teleost fish. Water Biol Secur 2:100139. 10.1016/j.watbs.2023.100139

[CR20] Chong GLW, Böhmert B, Lee LEJ, Bols NC, Dowd GC (2022) A continuous myofibroblast precursor cell line from the tail muscle of Australasian snapper (*Chrysophrys auratus*) that responds to transforming growth factor beta and fibroblast growth factor. In Vitro Cell Dev Biol Anim 58:922–935. 10.1007/s11626-022-00734-2 36378268 10.1007/s11626-022-00734-2 PMC9780137

[CR21] Claiborne JB, Edwards SL, Morrison-Shetlar AI (2002) Acid-base regulation in fishes: cellular and molecular mechanisms. J Exp Zool 293:302–319. 10.1002/jez.10125 12115903 10.1002/jez.10125

[CR22] Cuesta A, Meseguer J, Esteban MA (2011) Immunotoxicological effects of environmental contaminants in teleost fish reared for aquaculture. In: Stoytcheva M (ed) Pesticides in the modern world- Risks and Benefits. InTech. 10.5772/17430

[CR23] Dayeh VR, Schirmer K, Bols NC (2009) Ammonia-containing industrial effluents, lethal to rainbow trout, induce vacuolisation and neutral red uptake in the rainbow trout gill cell line, RTgill-W1. Altern Lab Anim 37:77–87. 10.1177/026119290903700111 19292578 10.1177/026119290903700111

[CR24] Dongre A, Weinberg RA (2019) New insights into the mechanisms of epithelial-mesenchymal transition and implications for cancer. Nat Rev Mol Cell Bio 20:69–84. 10.1038/s41580-018-0080-4 30459476 10.1038/s41580-018-0080-4

[CR25] Emenike EC, Iwuozor KO, Anidiobi SU (2022) Heavy Metal Pollution in Aquaculture: Sources, Impacts and Mitigation Techniques. Biol Trace Elem Res 200:4476–4492. 10.1007/s12011-021-03037-x 34813030 10.1007/s12011-021-03037-x

[CR26] Evans DH, Piermarini PM, Choe KP (2005) The multifunctional fish gill: dominant site of gas exchange, osmoregulation, acid-base regulation, and excretion of nitrogenous waste. Physiol Rev 85:97–177. 10.1152/physrev.00050.2003 15618479 10.1152/physrev.00050.2003

[CR27] FAO (2022) The State of World Fisheries and Aquaculture 2022. Towards Blue Transformation. FAO, Rome, Italy. 10.4060/cc0461en

[CR28] Fischer M, Belanger SE, Berckmans P, Bernhard MJ, Bláha L, Coman Schmid DE, Dyer SD, Haupt T, Hermens JLM, Hultman MT, Laue H, Lillicrap A, Mlnaříková M, Natsch A, Novák J, Sinnige TL, Tollefsen KE, von Niederhäusern V, Witters H, Županič A, Schirmer K (2019) Repeatability and Reproducibility of the RTgill-W1 Cell Line Assay for Predicting Fish Acute Toxicity. Toxicol Sci 169:353–364. 10.1093/toxsci/kfz057 30825313 10.1093/toxsci/kfz057 PMC6542334

[CR29] Fleurbaix E, Parant M, Maul A, Cossu-Leguille C (2022) Toxicity of lanthanides on various fish cell lines. Ecotoxicology 31:1147–1157. 10.1007/s10646-022-02574-y 35994187 10.1007/s10646-022-02574-y

[CR30] Franco ME, Sutherland GE, Lavado R (2018) Xenobiotic metabolism in the fish hepatic cell lines Hepa-E1 and RTH-149, and the gill cell lines RTgill-W1 and G1B: Biomarkers of CYP450 activity and oxidative stress. Comp Biochem Physiol C Toxicol Pharmacol 206–207:32–40. 10.1016/j.cbpc.2018.02.006 29496489 10.1016/j.cbpc.2018.02.006

[CR31] Gjessing MC, Aamelfot M, Batts WN, Benestad SL, Dale OB, Thoen E, Weli SC, Winton JR (2018) Development and characterization of two cell lines from gills of Atlantic salmon. Plos One 13:e0191792. 10.1371/journal.pone.0191792 10.1371/journal.pone.0191792 PMC5812586 29444101

[CR32] Greenwell MG, Sherrill J, Clayton LA (2003) Osmoregulation in fish. Mechanisms and clinical implications. Vet Clin North Am Exot Anim Pract 6:169–189. 10.1016/s1094-9194(02)00021-x 10.1016/s1094-9194(02)00021-x 12616839

[CR33] Gülden M, Seibert H (2005) Impact of bioavailability on the correlation between in vitro cytotoxic and in vivo acute fish toxic concentrations of chemicals. Aquat Toxicol 72:327. 10.1016/j.aquatox.2005.02.002 15848252 10.1016/j.aquatox.2005.02.002

[CR34] Gülden M, Seibert H (2007) The improvement of in vitro cytotoxicity testing for the assessment of acute toxicity in fish. Altern Lab Anim 35:39–46. 10.1177/026119290703500108 17411350 10.1177/026119290703500108

[CR35] Hernández-Moreno D, Blázquez M, Navas JM, Fernández-Cruz ML (2022a) Fish cell lines as screening tools to predict acute toxicity to fish of biocidal active substances and their relevant environmental metabolites. Aquat Toxicol 242:106020. 10.1016/j.aquatox.2021.106020 34844051 10.1016/j.aquatox.2021.106020

[CR36] Hernández-Moreno D, Navas JM, Fernández-Cruz ML (2022b) Short and long-term effects of nanobiomaterials in fish cell lines. Applicability of RTgill-W1. Chemosphere 309:136636. 10.1016/j.chemosphere.2022.136636 36181847 10.1016/j.chemosphere.2022.136636

[CR37] Herrmann H, Munick MD, Brettel M, Fouquet B, Markl J (1996) Vimentin in a cold-water fish, the rainbow trout: Highly conserved primary structure but unique assembly properties. J Cell Sci 109:569–578. 10.1242/jcs.109.3.569 8907703 10.1242/jcs.109.3.569

[CR38] Hsiung J, Zhu DH, Hinton DR (2015) Polarized Human Embryonic Stem Cell-Derived Retinal Pigment Epithelial Cell Monolayers Have Higher Resistance to Oxidative Stress-Induced Cell Death Than Nonpolarized Cultures. Stem Cell Transl Med 4:10–20. 10.5966/sctm.2014-0205 10.5966/sctm.2014-0205 PMC4275015 25411476

[CR39] Ivanova L, Fæste CK, Solhaug A (2023) Atlantic salmon gill epithelial cell line (asg-10) as a suitable model for xenobiotic biotransformation. Metabolites 13:771. 10.3390/metabo13060771 37367928 10.3390/metabo13060771 PMC10303838

[CR40] Jimeno-Romero A, Gwinner F, Müller M, Mariussen E, Soto M, Kohl Y (2021) Sea bass primary cultures versus rtgill-w1 cell line: Influence of cell model on the sensitivity to nanoparticles. Nanomaterials 11:3136. 10.3390/nano11113136 34835900 10.3390/nano11113136 PMC8620814

[CR41] Krogh A (2015) Osmotic regulation in aquatic animals. Cambridge University Press

[CR42] Lee LEJ, Dayeh VR, Schirmer K, Bols NC (2009) Applications and potential uses of fish gill cell lines: examples with RTgill-W1. In Vitro Cell Dev Biol Anim 45:127–134. 10.1007/s11626-008-9173-2 19184248 10.1007/s11626-008-9173-2

[CR43] Leguen II, Carlsson C, Perdu-Durand E, Prunet P, Pärt P, Cravedi JP (2000) Xenobiotic and steroid biotransformation activities in rainbow trout gill epithelial cells in culture. Aquat Toxicol 48:165–176. 10.1016/s0166-445x(99)00043-0 10686323 10.1016/s0166-445x(99)00043-0

[CR44] Lilius H, Sandbacka M, Isomaa B (1995) The use of freshly isolated gill epithelial cells in toxicity testing. Toxicol in Vitro 9:299–305. 10.1016/0887-2333(95)00010-6 20650091 10.1016/0887-2333(95)00010-6

[CR45] Mandal SC, Weidmann M, Albalat A, Carrick E, Morro B, MacKenzie S (2020) Polarized trout epithelial cells regulate transepithelial electrical resistance, gene expression, and the phosphoproteome in response to viral infection. Front Immunol 11:1809. 10.3389/fimmu.2020.01809 10.3389/fimmu.2020.01809 PMC7456818 32922394

[CR46] Markl J, Franke WW (1988) Localization of Cytokeratins in Tissues of the Rainbow-Trout - Fundamental Differences in Expression Pattern between Fish and Higher Vertebrates. Differentiation 39:97–122. 10.1111/j.1432-0436.1988.tb00086.x 2468546 10.1111/j.1432-0436.1988.tb00086.x

[CR47] Matsuo AY, Gallagher EP, Trute M, Stapleton PL, Levado R, Schlenk D (2008) Characterization of Phase I biotransformation enzymes in coho salmon (*Oncorhynchus kisutch*). Comp Biochem Physiol C Toxicol Pharmacol 147:78–84. 10.1016/j.cbpc.2007.08.001 17826357 10.1016/j.cbpc.2007.08.001 PMC2694037

[CR48] Matthee C, Brown AR, Lange A, Tyler CR (2023) Factors Determining the Susceptibility of Fish to Effects of Human Pharmaceuticals. Environ Sci Technol 57:8845–8862. 10.1021/acs.est.2c09576 37288931 10.1021/acs.est.2c09576 PMC10286317

[CR49] McCormick SD (2001) Endocrine control of osmoregulation in teleost fish. Am Zool 41:781–794. 10.1093/icb/41.4.781

[CR50] Mendez MG, Kojima SI, Goldman RD (2010) Vimentin induces changes in cell shape, motility, and adhesion during the epithelial to mesenchymal transition. Faseb J 24:1838–1851. 10.1096/fj.09-151639 20097873 10.1096/fj.09-151639 PMC2874471

[CR51] Moran D, Schleyken J, Flammensbeck C, Fantham W, Ashton D, Wellenreuther M (2023) Enhanced survival and growth in the selectively bred *Chrysophrys auratus* (Australasian snapper, tāmure). Aquaculture 563:738970. 10.1016/j.aquaculture.2022.738970

[CR52] Natsch A, Laue H, Haupt T, von Niederhäusern V, Sanders G (2018) Accurate prediction of acute fish toxicity of fragrance chemicals with the RTgill-W1 cell assay. Environ Toxicol Chem 37:931–941. 10.1002/etc.4027 29105821 10.1002/etc.4027

[CR53] Nelson JS (1984) Fishes of the world, 2nd edn. Wiley, New York

[CR54] OECD (2021). Organisation for economic co-operation and development. Test No. 249: OECD guidelines for the testing of chemicals; Test no. 249: Fish cell line acute toxicity - the RTgill-W1 cell line assay. 10.1787/c66d5190-en

[CR55] Olson KR (1998) Hormone metabolism by the fish gill. Comp Biochem Physiol A Mol Integr Physiol 119:55–65. 10.1016/S1095-6433(97)00406-6 11253819 10.1016/s1095-6433(97)00406-6

[CR56] Paparella M, Scholz S, Belanger S, Braunbeck T, Bicherel P, Connors K, Faßbender C, Halder M, Lillicrap A, Liska R, Schirmer K, Stoddart G, Thomas P, Walter-Rohde S (2021) Limitations and uncertainties of acute fish toxicity assessments can be reduced using alternative methods. Altex 38:20–32. 10.14573/altex.2006051 32970822 10.14573/altex.2006051

[CR57] Pumputis PG, Braley E, Hamilton ME, Dayeh VR, Lee LEJ, Bols NC (2022) Integrity and wound healing of rainbow trout intestinal epithelial cell sheets at hypo-, normo-, and hyper-thermic temperatures. J Therm Biol 103:103147. 10.1016/j.jtherbio.2021.103147 10.1016/j.jtherbio.2021.103147 35027200

[CR58] Reid NM, Proestou DA, Clark BW, Warren WC, Colbourne JK, Shaw JR, Karchner SI, Hahn ME, Nacci D, Oleksiak MF, Crawford DL, Whitehead A (2016) The genomic landscape of rapid repeated evolutionary adaptation to toxic pollution in wild fish. Science 354:1305–1308. 10.1126/science.aah4993 27940876 10.1126/science.aah4993 PMC5206662

[CR59] Roberts RJ, Ellis AE (2012) The Anatomy and physiology of teleosts. In: Roberts RJ (ed) Fish pathology, pp 17–61. 10.1002/9781118222942.ch2

[CR60] Sandbacka M, Christianson I, Isomaa B (2000) Gill epithelial cells as in vitro models in aquatic toxicology. Altern Lab Anim 28:457–460. 10.1177/026119290002800316 25419926 10.1177/026119290002800316

[CR61] Schirmer K (2006) Proposal to improve vertebrate cell cultures to establish them as substitutes for the regulatory testing of chemicals and effluents using fish. Toxicology 224:163. 10.1016/j.tox.2006.04.042 16765501 10.1016/j.tox.2006.04.042

[CR62] Schirmer K, Chan AGJ, Greenberg BM, Dixon DG, Bols NC (1997) Methodology for demonstrating and measuring the photocytotoxicity of fluoranthene to fish cells in culture. Toxicol in Vitro 11:107. 10.1016/s0887-2333(97)00002-7 20654301 10.1016/s0887-2333(97)00002-7

[CR63] Schirmer K, Tanneberger K, Kramer NI, Volker D, Scholz S, Hafner C, Lee LE, Bols NC, Hermens JLM (2008) Developing a list of reference chemicals for testing alternatives to whole fish toxicity tests. Aquat Toxicol 90:128. 10.1016/j.aquatox.2008.08.005 18829120 10.1016/j.aquatox.2008.08.005

[CR64] Scott J, Mortensen S, Minghetti M (2023) Alternatives to fish acute whole effluent toxicity (wet) testing: Predictability of RTgill-W1 cells and fathead minnow embryos with actual wastewater samples. Environ Sci Technol 57:13721–13731. 10.1021/acs.est.3c02067 37672649 10.1021/acs.est.3c02067

[CR65] Segner H (1998) Fish cell lines as a tool in aquatic toxicology. EXS 86:1–38. 10.1007/978-3-0348-8853-0_1 9949875 10.1007/978-3-0348-8853-0_1

[CR66] Shi F, McNabb P, Rhodes L, Holland P, Webb S, Adamson J, Immers A, Gooneratne R, Holland J (2012) The toxic effects of three dinoflagellate species from the genus Karenia on invertebrate larvae and finfish. N Z J Mar Freshwater Res 46:149–165. 10.1080/00288330.2011.616210

[CR67] Slattery O, Dahle MK, Sundaram AYM, Nowak BF, Gjessing MC, Solhaug A (2023) Functional and molecular characterization of the Atlantic salmon gill epithelium cell line ASG-10; a tool for gill research. Front Mol Biosci 10.1242879. 10.3389/fmolb.2023.1242879 10.3389/fmolb.2023.1242879 PMC10616884 37916189

[CR68] Smolowitz RM, Schultz ME, Stegeman JJ (1992) Cytochrome P4501A induction in tissues, including olfactory epithelium, of topminnows (*Poeciliopsis spp*.) by waterborne benzo[a]pyrene. Carcinogenesis 13:2395–2402. 10.1093/carcin/13.12.2395 1473249 10.1093/carcin/13.12.2395

[CR69] Solhaug A, Gjessing M, Sandvik M, Eriksen GS (2023) The gill epithelial cell lines RTgill-W1, from Rainbow trout and ASG-10, from Atlantic salmon, exert different toxicity profiles towards rotenone. Cytotechnology 75:63–75. 10.1007/s10616-022-00560-0 36713067 10.1007/s10616-022-00560-0 PMC9880101

[CR70] Stadnicka-Michalak J, Weiss FT, Fischer M, Tanneberger K, Schirmer K (2018) Biotransformation of Benzo[a]pyrene by three rainbow trout (Onchorhynchus mykiss) cell lines and extrapolation to derive a fish bioconcentration factor. Environ Sci Technol 52:3091–3100. 10.1021/acs.est.7b04548 29400055 10.1021/acs.est.7b04548

[CR71] Taju G, Abdul Majeed S, Nambi KSN, Sahul Hameed AS (2014) In vitro assay for the toxicity of silver nanoparticles using heart and gill cell lines of *Catla catla* and gill cell line of *Labeo rohita*. Comp Biochem Physiol C Toxicol Pharmacol 161:41–52. 10.1016/j.cbpc.2014.01.007 24524868 10.1016/j.cbpc.2014.01.007

[CR72] Tanneberger K, Knöbel M, Busser FJM, Sinnige TL, Hermens JLM, Schirmer K (2013) Predicting fish acute toxicity using a fish gill cell line-based toxicity assay. Ecotoxicol Environ Safety 47:1110–1119. 10.1021/es303505z 10.1021/es303505z 23227966

[CR73] Te Aika B, Liggins L, Rye C, Perkins EO, Huh J, Brauning R, Godfery T, Black MA (2023) Aotearoa genomic data repository: An āhuru mōwai for taonga species sequencing data. Mol Eco Res 00:1–14. 10.1111/1755-0998.13866 10.1111/1755-0998.13866 PMC11696480 37712601

[CR74] Vo NTK, Bender AW, Ammendolia DA, Lumsden JS, Dixon B, Bols NC (2015) Development of a walleye spleen stromal cell line sensitive to viral hemorrhagic septicemia virus (VHSV IVb) and to protection by synthetic dsRNA. Fish Shellfish Immun 45:83–93. 10.1016/j.fsi.2015.02.004 10.1016/j.fsi.2015.02.004 25701636

[CR75] Wellenreuther M, Le Luyer J, Cook D, Ritchie PA, Bernatchez L (2019) Domestication and temperature modulate gene expression signatures and growth in the Australasian snapper *Chrysophrys auratus*. G3 Genes|Genomes|Genetics 9:105–116. 10.1534/g3.118.200647 10.1534/g3.118.200647 PMC6325909 30591433

[CR76] Wilson JM, Laurent P (2002) Fish gill morphology: Inside out. J Exp Zool 293:192–213. 10.1002/jez.10124 12115897 10.1002/jez.10124

[CR77] Wootton RJ (1990) Ecology of teleost fishes (Fish and Fisheries Series 1). London: Chapman and Hall, p 404

[CR78] Xu X, Song Z, Li Z, Liu X, Feng Y, Wang W, Sun G, Yang J (2021) Establishment and characterization of a gill cell line from pearl gentian grouper (*Epinephelus lanceolatus*♂×*Epinephelus fuscoguttatus*♀) and its application in cadmium toxicology. Ecotoxicol Environ Safety 208:111614. 10.1016/j.ecoenv.2020.111614 33396134 10.1016/j.ecoenv.2020.111614

[CR79] Yue Y, Behra R, Sigg L, Fernandez Freire P, Pillai S, Schirmer K (2015) Toxicity of silver nanoparticles to a fish gill cell line: role of medium composition. Nanotoxicology 9:54–63. 10.3109/17435390.2014.889236 24621324 10.3109/17435390.2014.889236

[CR80] Yue Y, Li X, Sigg L, Suter MJ, Pillai S, Behra R, Schirmer K (2017) Interaction of silver nanoparticles with algae and fish cells: a side by side comparison. J Nanobiotechnology 15:16. 10.1186/s12951-017-0254-9 28245850 10.1186/s12951-017-0254-9 PMC5331694

[CR81] Zeng F, Sherry JP, Bols NC (2016) Use of the rainbow trout cell lines, RTgill-W1 and RTL-W1 to evaluate the toxic potential of benzotriazoles. Ecotoxicol Environ Safety 124:315–323. 10.1016/j.ecoenv.2015.11.003 26584462 10.1016/j.ecoenv.2015.11.003

[CR82] Zimmer AM, Perry SF (2022) Physiology and aquaculture: A review of ion and acid-base regulation by the gills of fishes. Fish 23:874–898. 10.1111/faf.12659

[CR83] Zurita J, Peso Ad, Rojas R, Maisanaba S, Repetto G (2019) Integration of fish cell cultures in the toxicological assessment of effluents. Ecotoxicol Environ Safety 176:309–320. 10.1016/j.ecoenv.2019.03.101 30951978 10.1016/j.ecoenv.2019.03.101

