# Supplementary material for: Isolation and characterisation of two epithelial-like cell lines from the gills of Chrysophrys auratus (Australasian snapper) and Oncorhynchus tshawytscha (Chinook salmon) and their use in aquatic toxicology
Source: In Vitro Cell Dev Biol Anim. 2024 Jul 10;61(5):548–60. doi: 10.1007/s11626-024-00941-z (PMC12246032; doi:10.1007/s11626-024-00941-z)
Supplement: Supplementary file 1 — Supplementary file1 (DOCX 6.40 MB) [file 11626_2024_941_MOESM1_ESM.docx]

**Supplementary Information**

**Methods:**

***Immunohistochemistry***

Gill fragments were dissected from freshly euthanised salmon and snapper by an overdose of anaesthetic (60 ppm of AQUI-S®; AQUI-S Ltd, Lower Hutt, New Zealand). Tissues were fixed in 10% neutral buffered formalin (NBF) (Thermo Fisher) for 24 h, then transferred to 70% ethanol for at least 24 h. Gill arches were then dissected and decalcified in 5% formic acid (Thermo Fisher) for 24 h to 1 week. Complete decalcification was tested using 2.5% ammonium hydroxide/2.5% ammonium oxalate (Sigma-Aldrich). After decalcification, the tissues were embedded into paraffin wax following a series of dehydration steps. Paraffin-embedded tissues were then processed into serial 4 µm sections with one slide stained with haematoxylin & eosin to assess the tissue architecture and identify primary and secondary lamella. Sections were deparaffinised and rehydrated, then antigens retrieved using tris-urea buffer with a pH of 9.5 at 95°C for 20 min (0.05 M Tris-HCL and 0.83 mM urea; both Thermo Fisher).

Primary antibodies targeting cytokeratin (1:250, mouse anti-pan cytokeratin PCK-26 monoclonal, C2562 Sigma-Aldrich), E-cadherin (1:50, rabbit anti-Cdh1 polyclonal, GTX125890 GeneTex), fibronectin (1:50, mouse anti-fibronectin IST-3 monoclonal, F0791, Sigma-Aldrich), and vimentin (1:100, rabbit anti-Vim polyclonal, GTX133061, GeneTex) were added to sections in a final volume of 20 µL with staining diluent (of 1% BSA (Gibco) and 0.05% Triton X-100 (Sigma-Aldrich) in tris-buffered saline (TBS; 0.05 M Tris-HCl, Thermo Fisher, 0.15 M NaCl, Labserve, South Africa, pH 7.6) at 4 °C overnight.

Sections were then washed three times with TBS, followed by incubation with 20 µL goat anti-mouse or goat anti-rabbit secondary antibody (1:500, Alexa Fluor™ 488, A11008 (mouse) A11020 (rabbit), Thermo Fisher) and DAPI (100 µM 4′,6-diamidino-2-phenylindole, D3571, Invitrogen) in staining diluent for 1 h at room temperature. Sudan black solution (20 µL of 0.1% solution in ethanol, Sigma-Aldrich) was added to each section for 25 min at room temperature followed by a 10 min wash in TBS. Stained sections were mounted in Slow Fade Gold (S36936, Invitrogen) overnight prior to imaging on an Olympus BX63 microscope using the U-FBNA filter cube (Ex 470–495 nm, emission 510–550 nm) and U-FUNA filter cube (excitation 360-370 nm, emission 420–460 nm) and cellSens Dimension software.


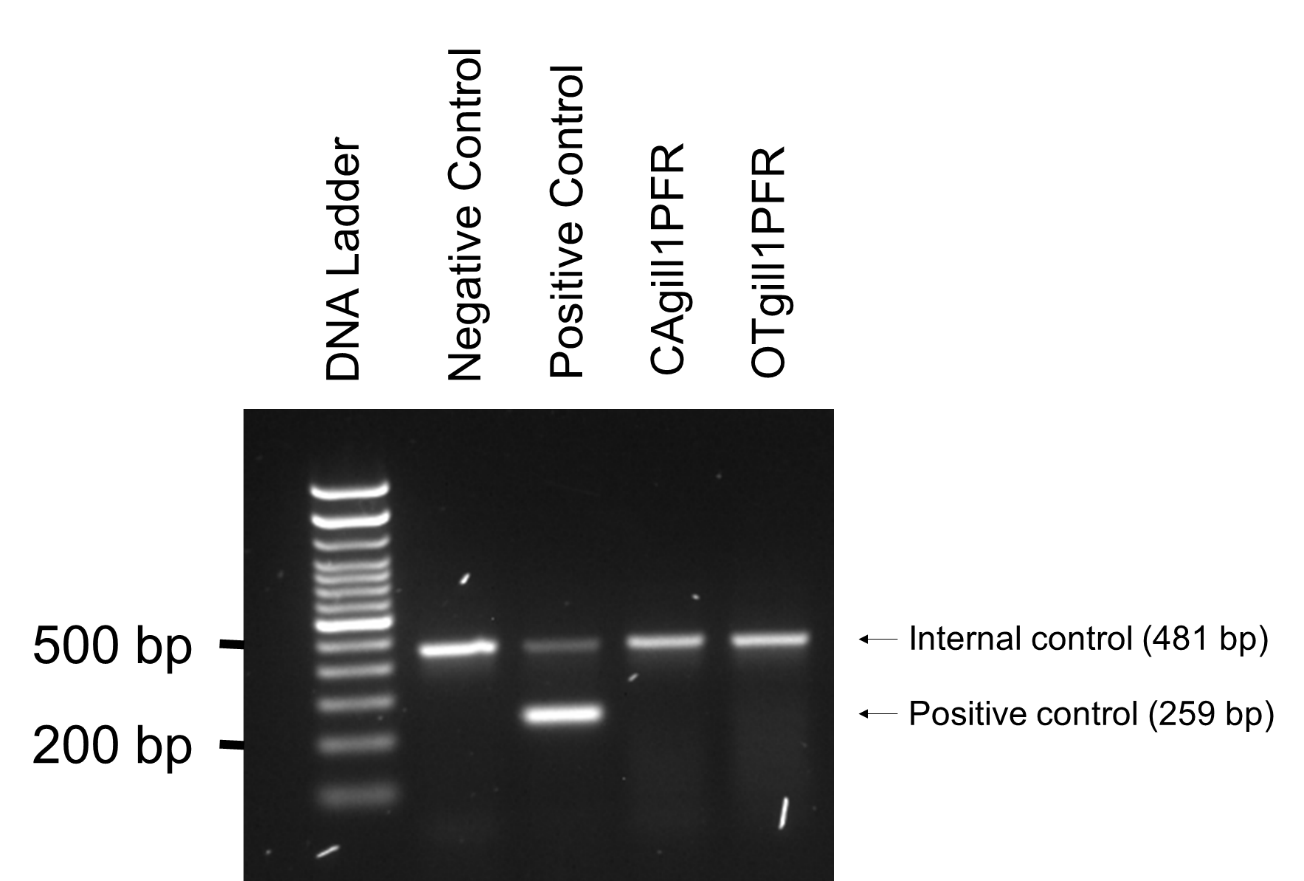


**Figure S1**: Agarose gel for detection of *Mycoplasma* sp. in OTgill1PFr and CAgill1PFR. The internal control band is visible across all samples at 481 bp. The band at 259 bp in the positive control, indicative of *Mycoplasma* contamination, is absent from CAgill1PFr and OTgill1PFR.


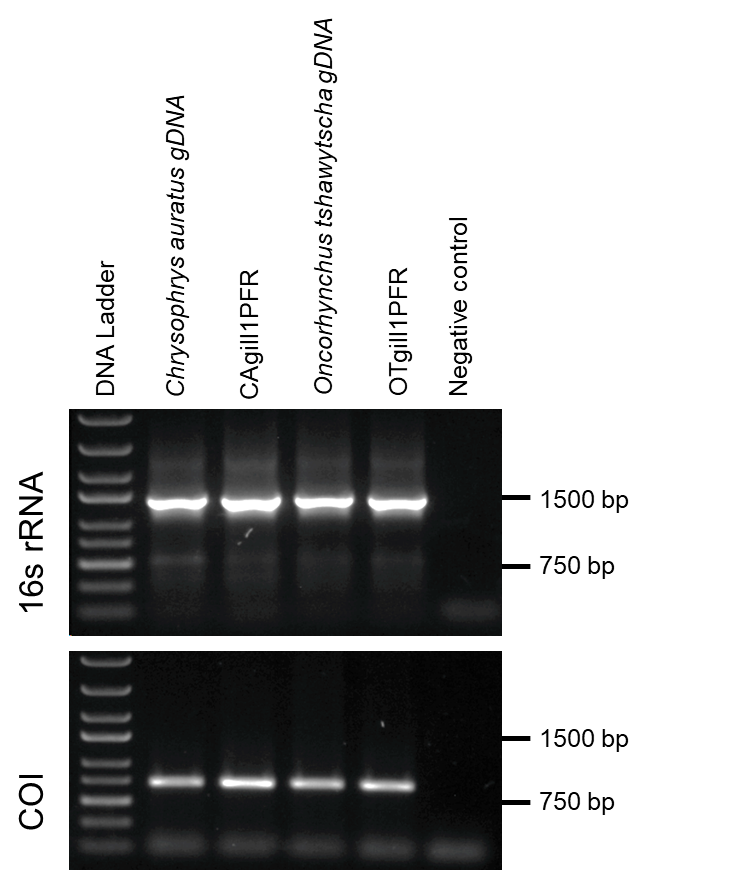


**A**

**B**

>COI_ *Oncorhynchus tshawytscha*

GGCACCCTCTATTTAGTATTTGGTGCCTGAGCCGGGATAGTAGGCACCGCCCTTAGTCTACTGATTCGGGCAGAACTAAGCCAGCCGGGCGCTCTTCTAGGGGATGACCAGATCTATAACGTGATCGTCACAGCCCATGCCTTCGTTATGATTTTCTTTATAGTTATGCCGATTATGATCGGAGGCTTTGGAAACTGATTAATTCCCCTAATGATCGGGGCCCCTGATATGGCATTCCCTCGAATAAATAACATAAGCTTCTGACTCCTTCCACCATCCTTTCTCCTCCTCCTATCTTCCTCTGGAGTTGAAGCCGGAGCTGGCACCGGGTGAACAGTCTACCCCCCTCTGGCCGGCAACCTAGCCCACGCAGGAGCCTCAGTTGATCTGACGATCTTCTCCCTTCATTTAGCCGGGATCTCCTCAATTTTAGGAGCCATTAATTTTATTACTACCATTATTAACATAAAACCCCCGGCTATCTCTCAGTACCAAACCCCACTTTTTGTTTGAGCTGTACTAGTTACTGCTGTCCTTCTACTACTCTCCCTCCCCGTTCTGGCAGCAGGCATTACTATGTTACTCACGGACCGAAATCTAAACACCACTTTCTTTGACCCGGCAGGCGGGGGAGATCCAATTTTATACCAGCACCTCTTTTGATTCTT

>16SrRNA_*Oncorhynchus tshawytscha* ATAGGGACTCTGGGAGAGGATTGCGCTGTTATCCCTAGGGTAACTCGGTCCGTTGATCGGCGTTTGCCGGATCATTTTTGGTCAGATATTCTGGTGCTTAGAGCTGTGGCTCTTGGCTGTGGGGGCAGTGCCCCCAGTCCACATGGGGGCTTAATTTTCCCCCGCGGTCGCCCCAACCAAAGACATATGGGCTAGGGGTCACTGCGTTTTTACTTGTTAATTCAAGGTTGTTTGACGTGATCTGCCTGGTGTCTAAAGCTCCATAGGGTCTTCTCGTCTTATGTGCTTATGTCCGCTTCTGCACGGGCAGATCAATTTCATTGACTTGAAAGAGGAGACAGCTAAGCCCTCGTGATGCCATTCATACAGGTCTTCATTTAAAAGACAAGTGATTGCGCTACCTTCGCACGGTCAAAATACCGCGGCCGTTAAACCCATAGTCACAGGGCAGGCGGGACCTCTTATGTTTTGATTTGCAAGAGGCGATGTTTTTGGTAAACAGGCGAGGCTTGTGTTTGCCGAGTTCCTTCTCTTTCTTTGGGTCTTTCCCTGGGGGCACTCCTGTGTGGGGTTAACGATTTATTAGTGTAGGTTTTTCTTGGTGTTTATTCTGGTCTACAGTTCCCTCTTGGTTTGGGTTCGTTATTTGTCGGGGGTGGGTCCGATCCGACTTACACATGTGCTGGGAGAGAGTCGTCCTCTCTTATTACTCATTCTAGCATAATCTCTTCCATGGGGGCATGGAACGGCTTAGTACGGTTAGGGGGGTGGGATTTCTTATCAGGATAAGAGGTTTATATCTGTCTGAGCTTTAACGCTTTCTGTGCAGGTGGCTGCTCTTAGGCCCACTGTAACAGGTTACCTTAGTAATTATGATCC

# Figure S2: (A) PCR amplification of 16S rRNA and COI genes from genomic (gDNA) extracted from the fins of *Chrysophrys auratus* (snapper) and *Oncorhynchus tshawytscha* (Chinook salmon) and cell lines CAgill1PFR and OTgill1PFR. Amplicons were sequenced and in vitro and in vivo sequences analysed for homology. (B) Sequences for COI and 16S rRNA for *Oncorhynchus tshawytscha. Chrysophrys auratus* sequences are available from the national infrastructure platform Genomics Aotearoa in New Zealand (<https://www.genomics-aotearoa.org.nz/data>).


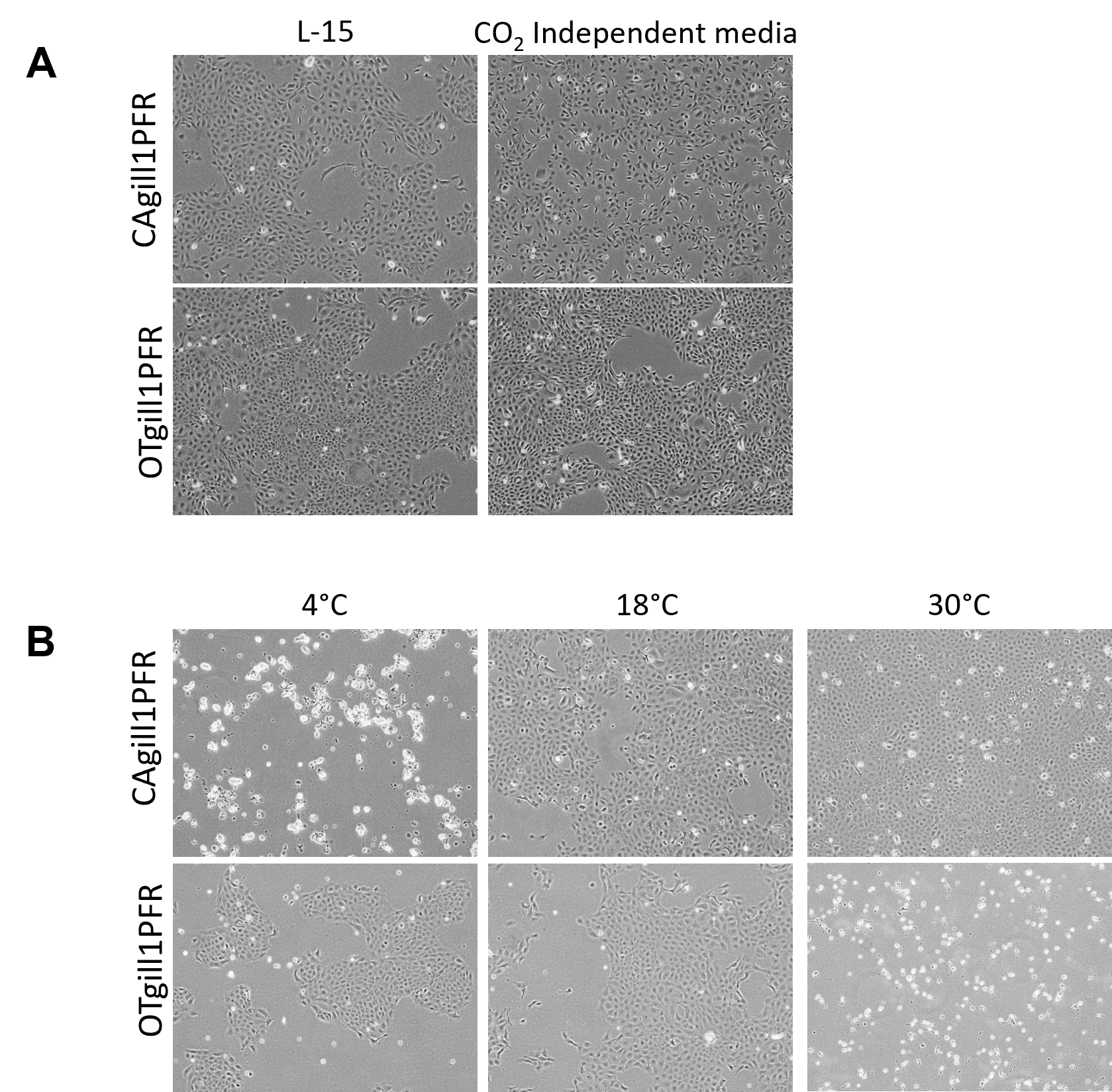


**Figure S3:** (A) Culture of CAgill1PFR and OTgillPFR in CO_2_ independent media causes a change in morphology/growth pattern. CAgill1PFR cells grow in isolation dispersed through the surface of the flask compared with culture in clusters/colonies in L-15 medium. OTgill1PFR cells appear to lose the distinct borders between cells seen during culture in L15. (B) After 4 days in culture, effects of temperature on OTgill1PFR and CAgill1PFR are apparent. At 4 °C CAgill1PFR cells have died, whereas OTgill1PFR cells have remained viable. At 30 °C, CAgill1PFR have proliferated, whereas OTgill1PFR have perished.


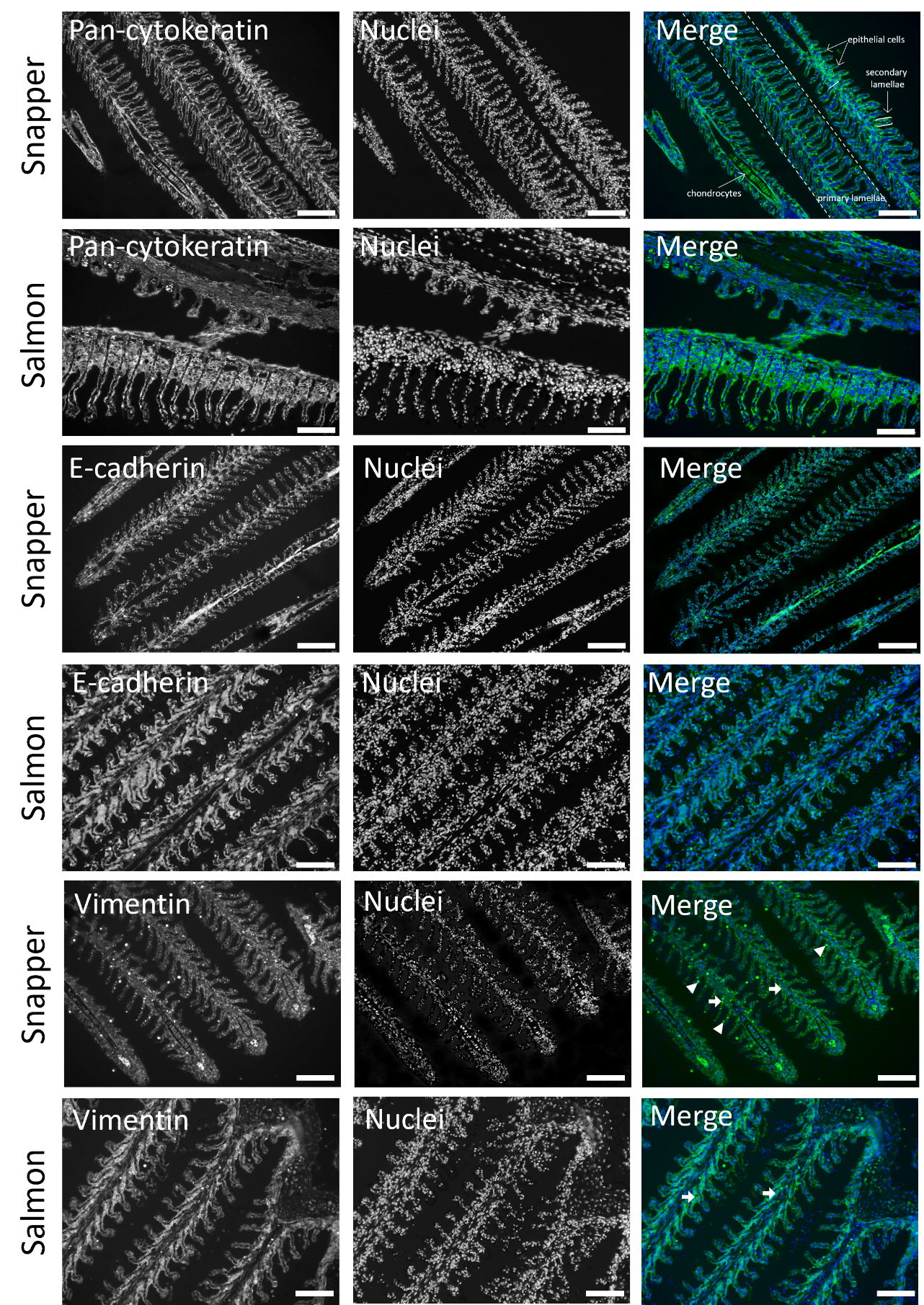


**Figure S4:** Immunohistochemistry detecting pan-cytokeratin, e-cadherin and vimentin in snapper and salmon gill tissue. Primary (dashed line) and secondary lamella (closed brackets) are identified in the merge image for snapper cytokeratin, as are chondrocytes and epithelial cells. Arrow heads highlight cells strongly expressing vimentin in secondary lamella. Arrows indicate vimentin-expressing cells at the base of secondary lamella. Nuclei are stained with DAPI. Scale bars = 100 µm.

**Figure S5**


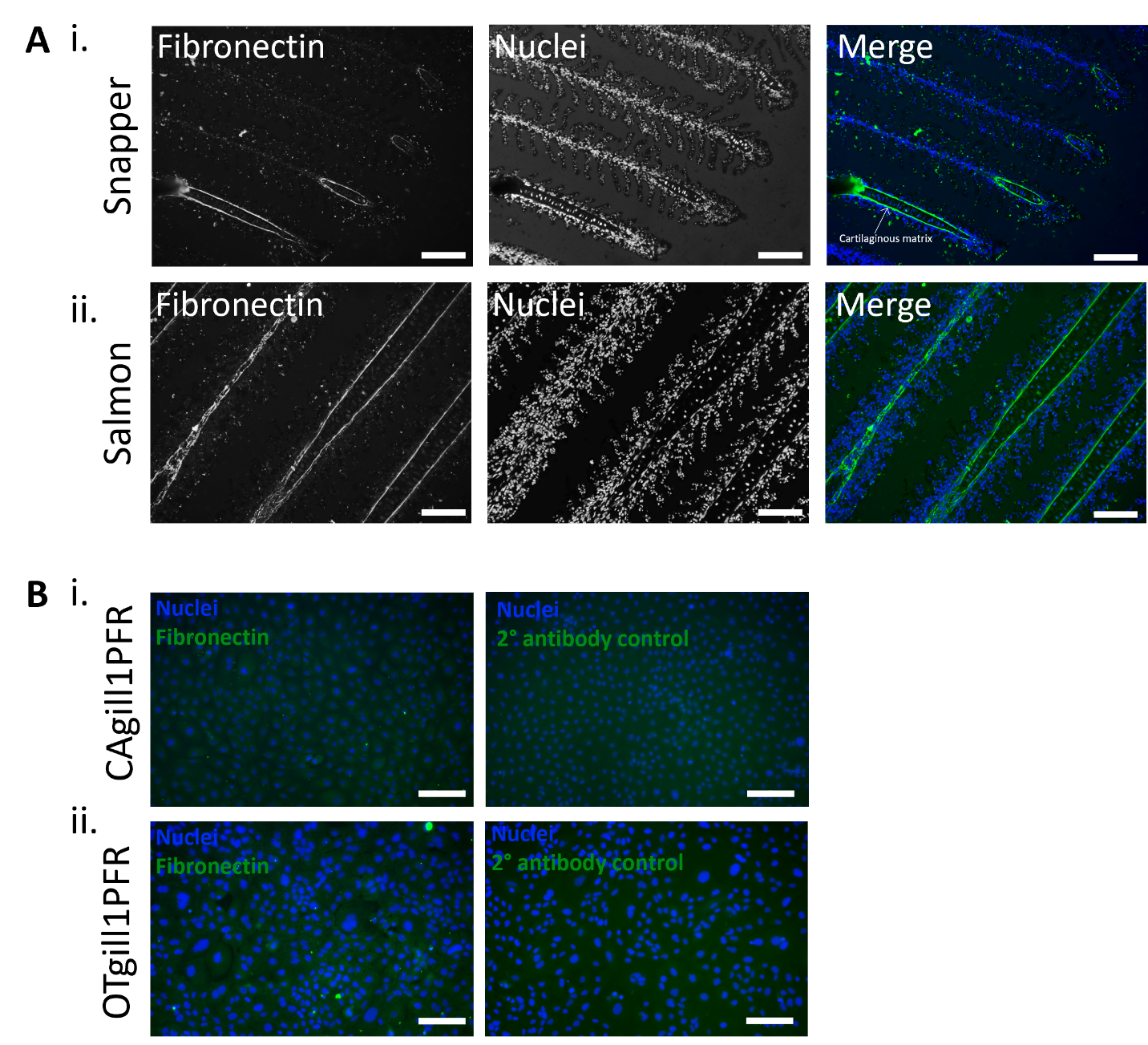


**Figure S5:** (A) Immunohistochemistry detection of fibronectin in snapper and salmon gill tissue. (B) Expression of fibronectin could not be detected in CAgill1PFR and Otgill1PFR. Cells with secondary antibody only serve as a control. Nuclei are stained with DAPI. Scale bars = 100 µm.
